# Modulation of Enzyme-Catalyzed Synthesis of Prostaglandins by Components Contained in Kidney Microsomal Preparations

**DOI:** 10.3390/molecules27010219

**Published:** 2021-12-30

**Authors:** Hyoung-Woo Bai, Jina Yu, Yue Wang, Pan Wang, Bao Ting Zhu

**Affiliations:** 1 Shenzhen Key Laboratory of Steroid Drug Discovery and Development, School of Life and Health Sciences and School of Medicine, The Chinese University of Hong Kong, Shenzhen 518172, China; hbai@kaeri.re.kr (H.-W.B.); genetic99@gmail.com (J.Y.); wangyue@cuhk.edu.cn (Y.W.); wangpan@cuhk.edu.cn (P.W.); 2 Advanced Radiation Technology Institute (ARTI), Korea Atomic Energy Research Institute (KAERI), Jeongeup-si 580-185, Korea; 3 Shenzhen Bay Laboratory, Shenzhen 518055, China

**Keywords:** cyclooxygease, kidney microsomes, arachidonic acid, prostaglandins

## Abstract

In the kidney, prostaglandins formed by cyclooxygenase 1 and 2 (COX-1 and COX-2) play an important role in regulating renal blood flow. In the present study, we report our observations regarding a unique modulatory effect of renal microsomal preparation on COX-1/2-mediated formation of major prostaglandin (PG) products in vitro. We found that microsomes prepared from pig and rat kidneys had a dual stimulatory–inhibitory effect on the formation of certain PG products catalyzed by COX-1 and COX-2. At lower concentrations, kidney microsomes stimulated the formation of certain PG products, whereas at higher concentrations, their presence inhibited the formation. Presence of kidney microsomes consistently increased the *K*_m_ values of the COX-1/2-mediated reactions, while the *V*_max_ might be increased or decreased depending on stimulation or inhibition observed. Experimental evidence was presented to show that a protein component present in the pig kidney microsomes was primarily responsible for the activation of the enzyme-catalyzed arachidonic acid metabolism leading to the formation of certain PG products.

## 1. Introduction

Cyclooxygenase 1 and 2 (COX-1 and COX-2) can convert arachidonic acid (AA) into prostaglandins H_2_ (PGH_2_), which is further converted to various prostaglandins (PGs), thromboxanes (TX) and hydroxyeicosateraenoic acids (HETE). These autacoids have many important biological functions in the body, via activation of specific membrane receptors [1,2]. The COX enzymes have two distinct catalytic activities: one for cyclooxygenation that converts AA to prostaglandins G_2_ (PGG_2_), and one for peroxidation that further transforms PGG_2_ to PGH_2_ [3,4]. Two COX isoforms, namely COX-1 and COX-2, have been identified [5,6,7,8], and they share ~60% overall sequence similarity but with much higher sequence homology in their catalytic regions [5,9]. COX-2, which is strongly induced by various mitogens, plays a more important role under certain pathological conditions such as inflammation, whereas COX-1, which is stably expressed in many tissues, mostly functions as a house-keeping enzyme [9,10]. These two COX enzymes are localized intracellularly to the luminal surfaces of the endoplasmic reticulum and the inner and outer membranes of the nuclear envelope [11].

In the mammalian kidney, PGs are mediators implicated in many important (patho)physiological processes [12,13]. COX-1 is abundantly expressed in the collecting ducts of the medulla [14,15], and is often considered a constitutively expressed enzyme, involved in the maintenance of normal physiological functions of the kidney, such as maintenance of water and salt balance [12]. However, COX-1 expression can also be altered under certain pathological conditions, such as glomerulonephritis [16]. COX-2 has rather different distribution and function in the kidney [13,17,18]. In the absence of stimulation, COX-2 is usually present at low but detectable levels in cells including the *macula densa* of the cortex, its adjacent cortical thick ascending limbs, and the lipid-laden medullary interstitial cells [13,19]. Increased COX-2 expression is usually seen in certain renal disorders such as obstructive nephropathy, glomerular diseases and Bartter’s syndrome [13,20,21,22,23,24].

In the present study, we report for the first time that certain components contained in the pig kidney microsomal fraction have a dual stimulatory–inhibitory effect on the production of certain PGs catalyzed by COX-1 and COX-2. At low concentrations, the presence of the pig kidney microsomes stimulated the COX-mediated formation of major PG products (not all PG products), but at higher concentrations, the presence of the pig kidney microsomes consistently exerted an inhibitory effect on COX-mediated formation of various PG products.

## 2. Results and Discussions

To test the in vitro enzymatic conversion of [^14^C]AA to PG products by COX-1 and COX-2, we adopted the assay conditions and procedures previously established in our laboratory [25]. The identity of these PG products was determined initially by comparing their retention times on the HPLC chromatographs with standard compounds, and then followed by LC-MS/MS analysis to confirm their structures as described in our earlier study [25]. After incubation of [^14^C]AA with COX-1 or COX-2, several major radioactive AA metabolites were detected, which included prostaglandin F_2α_ (PGF_2α_), prostaglandin E_2_ (PGE_2_), prostaglandin D_2_ (PGD_2_), and 12-hydroxyheptadecatrienoic acid (12-HHT). The minor radioactive AA metabolites included 5-HETE, 11-HETE, 12-HETE, 15-HETE, and TXB_2_, but TXA_2_ was not detected.

Here it is of note that PGH_2_ is a well-known direct product of COX-1/2-mediated AA metabolism, which can undergo non-enzymatic rearrangement to form 12-HHT [26,27]. In addition, PGH_2_ can also be enzymatically converted to PGE_2_, PGD_2_ and PGF_2α_ in the presence of the terminal synthases. We observed in this study and in our earlier studies [25,28] that PGE_2_, PGD_2_ and PGF_2α_ could be directly formed in vitro when [^14^C]AA was used as substrate and the partially purified COX-1 and COX-2 as the enzymes (without deliberately adding any terminal synthases in the reaction mixtures). Similar observations were also made in earlier studies by other investigators showing that PGE_2_ and PGD_2_ were detected when purified COX proteins were used as the enzyme source for the in vitro reactions [29,30]. These observations may have two possible explanations: One possibility is that there might be small amounts of PG terminal enzymes contained in the partially purified COX enzyme preparations. The COX-1 and COX-2 used in the present study were obtained from commercial sources, and their purity was 95% or 70%, respectively. Our observations from this study and from our earlier study [3] both showed the different ratios of 12-HHT to PGE_2_ for COX-1 and COX-2, which might be related to the different purities of the two enzyme preparations, and the COX enzyme preparations may contain other enzymes which have the terminal synthase activity. Another possibility is that some of the PGs (other than PGH_2_) might be formed non-enzymatically in vitro in the absence of the downstream synthases. This suggestion has also been made in some earlier studies [30,31]. As already mentioned above, the non-enzymatic conversion of PGH_2_ to 12-HHT is already known [26,27]. Theoretically, the non-enzymatic conversion of PGH_2_ to PGE_2_ only involves the breakdown of an unstable cyclic O–O bond in the PGH_2_ molecule (between C–O–O–C) via chemical reduction, which, in fact, has a good possibility of happening as there was GSH (1 mM) present in the in vitro reaction system used in this study.

**Figure 1:**
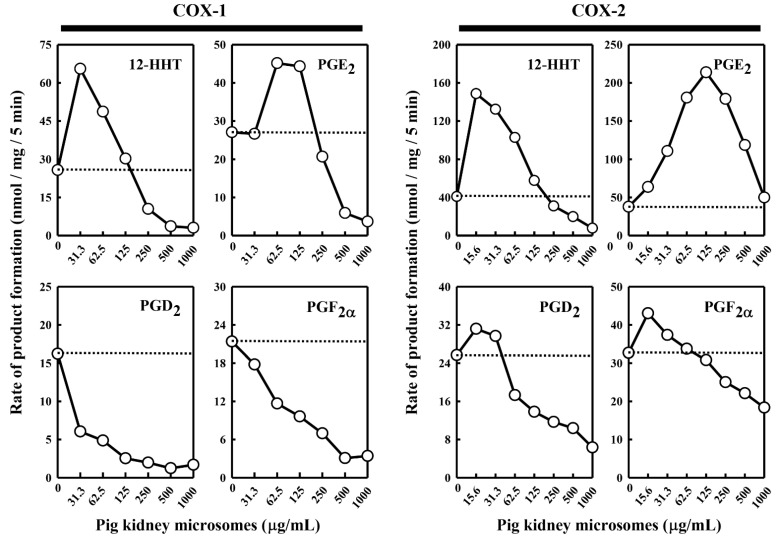
Effect of the pig kidney microsomes on COX-1 and COX-2-mediated conversion of [^14^C]AA to major PG products. The incubation mixture consisted of seven different concentrations (0, 31.3, 62.5, 125, 250, 500 and 1000 μg protein/mL) of the pig kidney microsomes, 20 μM [^14^C]AA (0.2 μCi) as substrate, 0.5 μg/mL COX-1 or 0.97 μg/mL COX-2 as the enzyme, 10 mM EDTA, 1 mM reduced glutathione, and 1 μM hematin in 200 μL of 100 mM Tris-HCl buffer, pH 7.4. The incubations were carried out at 37 °C for 5 min. The dotted line shows the control catalytic activity of COX-1 or COX-2 in the absence of the pig kidney microsomes. Each value is the mean of duplicate measurements, with small replicate variations (usually well below 10%).

Under the suitable conditions for assaying the in vitro [^14^C]AA metabolism by COX-1 and COX-2, we first chose to determine the ability of the pig kidney microsomes to modulate the enzymatic reactions. We found that when the pig kidney microsomes were incubated alone with 20 μM [^14^C]AA in the absence of COX-1 or COX-2, no appreciable AA metabolism was detected (data not shown), probably due to the relatively small amount of microsomal protein used. However, when the pig kidney microsomes were added to the COX-1 or COX-2 reaction mixtures, the COX-1/2-mediated formation of major PG products was altered, and the change was rather complex depending on the individual PG products formed and the concentrations of the kidney microsomal proteins present (Figure 1). 

In the case of COX-1-mediated reactions, formation of 12-HHT and PGE_2_ increased (with maximal increases by 260% and 167%, respectively) when the pig kidney microsomes were present at relatively low concentrations (≤125 μg/mL) (Figure 1). However, when higher microsomal protein concentrations were present, a concentration-dependent reduction of their formation was observed. In comparison, formation of PGF_2α_ and PGD_2_ was uniformly decreased in a concentration-dependent manner when different concentrations of the kidney microsomal proteins were present.

In the case of COX-2-mediated reactions, addition of the pig kidney microsomes at protein concentrations below 125 μg/mL produced a concentration-dependent stimulation of PGE_2_ formation, with a maximal increase by 575% (Figure 1). However, when the microsomal protein concentrations were higher, a concentration-dependent reduction of PGE_2_ formation started to occur. Similarly, when the pig kidney microsomal proteins were present at a very low concentration (15.6 μg/mL), formation of PGF_2α_, PGD_2_, and 12-HHT increased, with maximal increases by 134%, 124% and 370%, respectively. However, when higher concentrations of the kidney microsomal proteins were present (>15.6 μg/mL), a concentration-dependent reduction of their formation started to occur.

**Figure 2:**
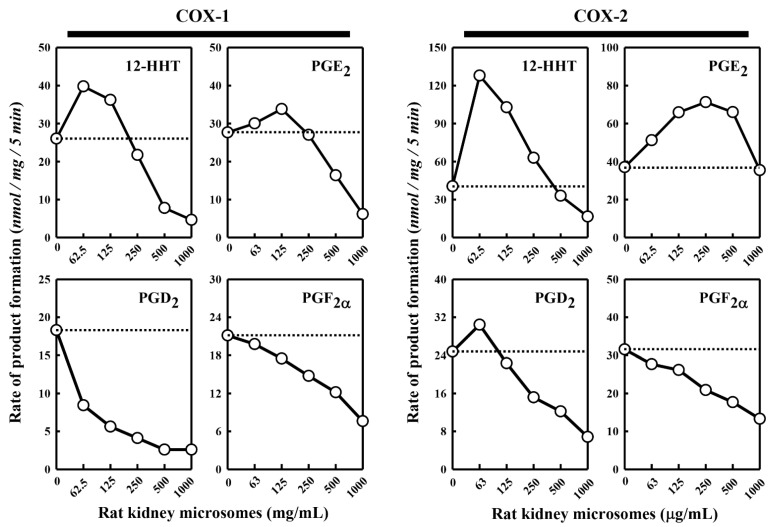
Effect of the rat kidney microsomes on COX-1 and COX-2-mediated conversion of [^14^C]AA to major PG products. The incubation mixture consisted of six different concentrations (0, 62.5, 125, 250, 500 and 1000 μg protein/mL) of the rat kidney microsomes, 20 μM [^14^C]AA (0.2 μCi) as substrate, 0.5 μg/mL COX-1 or 0.97 μg/mL COX-2 as the enzyme, 10 mM EDTA, 1 mM reduced glutathione, and 1 μM hematin in 200 μL of 100 mM Tris-HCl buffer, pH 7.4. The incubations were carried out at 37 °C for 5 min. The dotted line shows the control catalytic activity of COX-1 or COX-2 in the absence of the pig kidney microsomes. Each value is the mean of duplicate measurements, with small replicate variations (usually well below 10%).

Besides the pig kidney microsomes, we also tested the effect of the rat kidney microsomes on COX-1/2-mediated formation of representative PG products. We observed a similar dual stimulatory–inhibitory effect on COX-1/2-mediated formation of certain PG products (Figure 2). In the case of COX-1, the maximal stimulation of 12-HHT and PGE_2_ formation by the rat kidney microsomes was 153% and 122%, respectively, while the formation of PGD_2_ and PGF_2α_ was uniformly inhibited. Similarly, in the case of COX-2, the maximal stimulation of 12-HHT, PGE_2_ and PGD_2_ formation is 316%, 192% and 123%, respectively, by the rat kidney microsomes, whereas the formation of PGF_2α_ was inhibited. Overall, the rat kidney microsomes appeared to be less efficacious and less potent compared to the pig kidney microsomes.

For comparison, we also tested the effect of rat and human liver microsomes on COX-1/2-mediated PG formation. Again, we only observed a very small stimulatory–inhibitory effect on COX-2-mediated formation of PGE_2_ (data now shown). The maximal stimulation of COX-2-mediated formation of PGs was less than 20% above the control, but a strong inhibitory effect was observed when higher concentrations of the rat and human liver microsomes were present. Based on these observations, it appeared that the stimulatory component contained in the microsomal fraction was more richly contained in the kidney but not in the liver. On the contrary, the inhibitory component might be uniformly present in both kidney and liver microsomes. Moreover, the component contained in the kidney microsomes that stimulated the COX-1/2 catalytic activity was different from the component that inhibited the COX-1/2 catalytic activity. 

**Figure 3:**
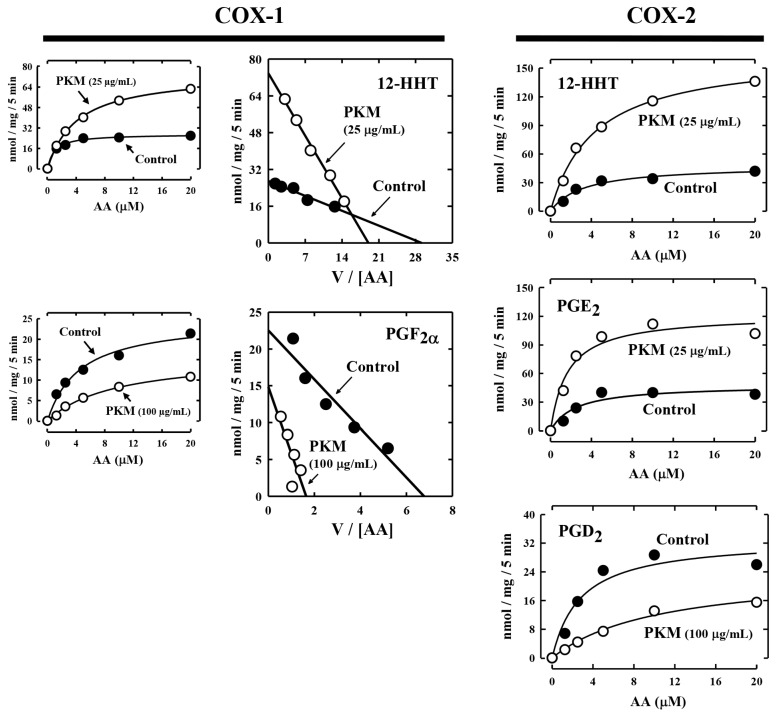
Representative Michaelis–Menten curves and Eadie–Hofstee plots for COX-1 and COX-2-mediated production of representative PG products in the presence of pig kidney microsomes. The incubation mixture consisted of [^14^C]AA (0.2 μCi) at indicated concentrations as substrate, 0.5 μg/mL COX-1 or 0.97 μg/mL COX-2 as the enzyme, 10 mM EDTA, 1 mM reduced glutathione, 1 μM hematin, and with or without pig kidney microsomes (at 25 or 100 μg protein/mL concentrations) in 200 μL of 100 mM Tris-HCl buffer, pH 7.4. Incubations were carried out at 37 °C for 5 min. Each data point is the mean of duplicate determinations. Kinetic parameters (*K*_m_ and *V*_max_) for the formation of major AA metabolites are summarized in Table 1. PKM stands for pig kidney microsomes.

To determine whether the pig kidney microsomes alter the *K*_m_ and *V*_max_ values of COX-1 and COX-2-mediated reactions, we analyzed the effect of pig kidney microsomes on COX-1/2-mediated formation of representative PG products. Some of the kinetic parameters (*K*_m_ and *V*_max_ values) are summarized in Table 1 and Figure 3. Changes in the kinetic values differed depending on the final products formed. In the case of COX-1, the *K*_m_ and *V*_max_ values for 12-HHT formation were both increased when the pig kidney microsomes were present. For PGF_2α_, the *V*_max_ value decreased by pig kidney microsomes, but its *K*_m_ value was markedly increased.

In the case of COX-2-mediated formation of 12-HHT and PGE_2_, their *V*_max_ values increased by the pig kidney microsomes, and their *K*_m_ values were also increased (Figure 3, Table 1). For PGD_2_ formation, its *K*_m_ was markedly increased, while its *V*_max_ was slightly decreased. Based on these observations, it was apparent that the presence of pig kidney microsomes would consistently increase the *K*_m_ values of COX-1/2-mediated formation of various PG products, regardless of whether it exerted a stimulatory effect or inhibitory effect. This implies that the presence of the pig kidney microsomes would somewhat reduce the apparent binding affinity of the COX-1 and COX-2 enzymes with their substrate arachidonic acid.

**Figure 4:**
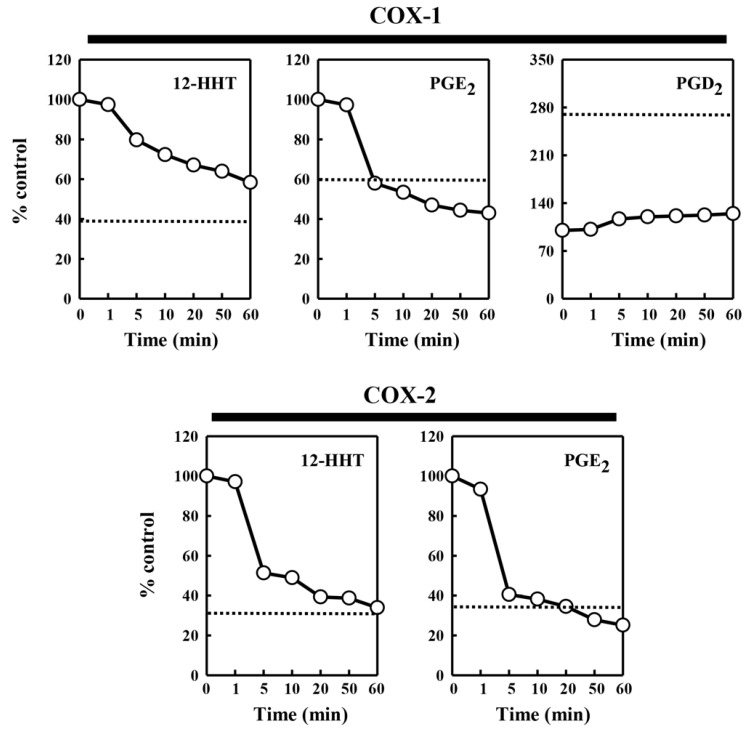
Effect of chymotrypsin pretreatment of the pig kidney microsomes on their ability to modulate the COX-1 and COX-2 catalytic activity. The incubation mixture consisted of 30 μg protein/mL of the pig kidney microsomes (pretreated with chymotrypsin), 20 μM [^14^C]AA (0.2 μCi) as a substrate, 0.5 μg/mL COX-1 and 0.97 μg/mL COX-2 as the enzyme in 10 mM EDTA, 1 mM reduced glutathione, and 1 μM hematin in 200 μL of 100 mM Tris-HCl buffer, pH 7.4. For the formation of PGE_2_ catalyzed by COX-1, the pig kidney microsomes (at 60 μg protein/mL, pretreated with chymotrypsin) were added. The incubations were carried out at 37 °C for 5 min. Chemotrypsin pretreatment of the pig kidney microsomes lasted 0, 0.5, 5, 10, 20, 40, and 60 min. Each value is the mean of duplicate measurements, with small replicate variations (usually well below 10%). The dotted line shows the relative level of the COX-2 catalytic activity in the absence of the pig kidney microsomes.

To probe whether the protein or lipid components contained in the pig kidney microsomes are responsible for the modulatory effect on COX-mediated formation of different PG products, we tested the effect of chymotrypsin (at 0.08 U), a common protease with broad substrate specificity, on the ability of the pig kidney microsomes to modulate the catalytic activity of COX-1 and COX-2. The data are summarized in Figure 4. After 5 min pre-incubation of the pig kidney microsomes with chymotrypsin, the microsomal stimulation of the COX-mediated 12-HHT and PGE_2_ formation was almost completely abolished. However, the microsomal inhibition of COX-1-mediated PGD_2_ formation was not affected by pre-incubation of the microsomes with chymotrypsin. This observation suggested that a protein component, but not a lipid component, contained in the pig kidney microsomes was primarily responsible for the stimulation of PG formation. In addition, when chymotrypsin was directly added to the COX-1/2-mediated reaction mixtures (in the absence of pig kidney microsomes), no similar effect on COX-1/2-mediated PG formation was observed (data not shown). 

The exact nature of this protein component contained in pig kidney microsomes and the mechanism of its activation of PG synthesis remain to be elucidated. It is speculated that the protein component contained in kidney microsomes may have terminal synthase activities that can directly facilitate the metabolic conversion of PGH_2_ to other PG products, or there may be a non-enzyme protein that can modulate the catalytic activities of the COX-1/2 and/or the terminal synthases. Here it is of interest to note that when superoxide dismutase (SOD, at 100 U/mL, obtained from Sigma-Aldrich) was added to the reaction mixture, a small increase in PGE_2_ formation (by approximately 50% above the control) was observed, but no appreciable inhibitory effect was seen (data not shown). This result likely suggested that the stimulatory effect was partially attributable to SOD, which is known to be richly contained in the kidney microsomes [32].

As for the chemical nature of the microsomal component that inhibited the COX-1/2 catalytic activity, it was apparent that a non-protein component likely was involved because the microsomal inhibition of COX-1-mediated PGD_2_ formation was not affected by pre-incubation of the microsomes with chymotrypsin. In addition, we found that when the pig kidney microsomes were boiled and then added to the incubation mixture, the stimulatory effect of the pig kidney microsomes on PG formation was abolished (data not shown), which is consistent with the observations with the chymotrypsin pretreatment results. However, the inhibitory effect on PG formation was also mostly abolished by boiling the pig kidney microsomes, which might be because pretreatment with a high temperature may also effectively destroy the key non-protein components contained in the kidney microsomes that are responsible for the inhibition of the enzyme-mediated PG formation.

In summary, the results of the present work suggest that the pig kidney microsomes have a dual stimulatory–inhibitory effect on the formation of certain PG products catalyzed by COX-1 and COX-2. At lower concentrations, the pig kidney microsomes stimulate PG formation whereas at higher concentrations, they inhibit PG formation. Enzyme kinetic analysis indicates that the *K*_m_ and *V*_max_ values of the COX-1/2-mediated reactions are altered by the pig kidney microsomes. It appears that some of the protein components contained in the pig kidney microsomes are primarily responsible for the observed stimulation of COX-mediated PG formation.

## 3. Materials and Methods

### 3.1. Chemicals

[^14^C]Arachidonic acid ([^14^C]AA, specific radioactivity of 53 Ci/mol) was purchased from PerkinElmer (Boston, MA, USA). Hematin, chymotrypsin, reduced glutathione (GSH) and oxidized glutathione (GSSG) were purchased from Sigma-Aldrich (St. Louis, MO, USA). COX-1, COX-2, PGE_2_, PGF_2α_, PGD_2_ and 12-hydroxy-5Z,8E,10E-heptadecatrienoic acid (12-HHT) were purchased from Cayman Co. (Ann Arbor, MI, USA).

### 3.2. Assay of COX-1 and COX-2 Catalytic Activity

To assay the COX-1 and COX-2 catalytic activity, the incubation mixture (added to an Eppendrof tube) consisted of 20 μM [^14^C]AA (0.2 μCi) as substrate, COX-1 or COX-2 as the enzyme (0.5 μg/mL or 0.97 μg/mL, respectively), 10 mM EDTA, 1 mM reduced glutathione, 1 μM hematin, and microsomal proteins in 200 μL of 100 mM Tris-HCl buffer, pH 7.4. The reaction was incubated at 37 °C for 5 min and terminated by addition of ice-cold 0.5 M HCl (15 μL) to each tube. Ethyl acetate (600 μL) was added immediately, and the products were extracted by vigorous voltexing, and centrifuged for 1 min in a microcentrifuge. The upper organic layer was transferred to another clean tube and dried under a stream of N_2_ gas. The dried metabolites in the vials were re-dissolved in acetonitrile and analyzed by high performance liquid chromatography (HPLC) (Agilent, Santa Clara, CA, USA) for metabolite composition. 

The HPLC system consisted of a Waters 2695 solvent delivery system, a Waters 2487 UV-detector, and an IN/US β-RAM radioactive detector, coupled with a C18 column (Atlantis, 4.6 × 150 mm) for separation. The elution of the AA metabolites included a linear gradient from 93% solvent A (25% acetonitrile in water containing 0.01% acetic acid) and 7% solvent B (100% acetonitrile containing 0.01% acetic acid) to 14% solvent A and 86% solvent B over a 27-min period at a flow rate of 1 mL/min. The gradient was then changed to 100% solvent A over a 3-min period at a flow rate of 1 mL/min. The radioactive fractions were detected using an IN/US β-RAM inline radioactive detector, while the nonradioactive co-eluting standards were detected at 200 nm.

### 3.3. Preparation of Microsomes

Fresh pig liver and kidney samples were obtained from a local meat plant. The collected tissues were snap-frozen in liquid nitrogen for transport to the laboratory for storage. On the day of preparation of microsomes, kidney samples were first thawed at room temperature and then rinsed with ice-cold normal saline. Connective tissues were removed with a pair of sharp surgery scissors. Tissues were then minced in 3 volumes of an ice-cold solution (pH 7.4) containing 0.05 M Tris-HCl and 1.15% KCl and then homogenized with a Tri-R homogenizer (model K41) for 2 to 3 min followed by a Teflon homogenizer (DuPont, Wilmington, DE, USA) for another 2 to 3 min. Tissue homogenates were centrifuged at 9000× *g* for 10 min, and supernatants were pooled and filtered through two layers of cheesecloth to remove lipid clots. The filtrates were then recentrifuged at 105,000× *g* (4 °C) for 90 min. The resulting pellets were the microsomal fraction, and the supernatants were the cytosolic fraction. The microsomal pellets were resuspended in 3 volumes of the washing buffer, and then centrifuged again at 105,000× *g* (4 °C) for 60 min. The supernatant was discarded, and the microsomal pellet was suspended in 250 mM sucrose. Aliquots of each cytosolic preparation and microsomal fraction were stored separately in small vials at −80 °C. The protein concentration was determined by the Bio-Rad protein assay kit (Bio-Rad, Hercules, CA, USA) with bovine serum albumin as standard. Using the same method and procedures as described above, the microsomal fractions from the rat kidney, rat liver, and human liver samples were also prepared in this study.

### 3.4. Treatment of Pig Kidney Microsomes with Chymotrypsin

Chymotrypsin was used to digest pig kidney microsomal proteins. Ten units of chymotrypsin were added to a solution containing 600 μg of the pig kidney microsomal proteins and incubated for different lengths of time at 37 °C. The pig kidney microsomes (at 30 μg protein/mL) were used for assaying their modulatory effect on the COX-1/2 catalytic activity, and the reaction mixture contained the same reagents as described above. 

### 3.5. Determination of Kinetic Parameters (K*_m_* and V*_max_*)

To determine the kinetic parameters (*K*_m_ and *V*_max_) of COX-1/2-mediated formation of representative PG products, selected concentrations of [^14^C]AA were used as substrate in the absence or presence of the pig kidney microsomal proteins. To determine the *K*_m_ (μM) and *V*_max_ (pmol/mg protein/min) values, the Michaelis–Menten curves were analyzed using the SigmaPlot 8.0 software, and the Eadie–Hofstee plots were also drawn.

## Figures and Tables

**Table 1:** Kinetic parameters (*K*_m_ and *V*_max_ values) for COX-1/2-catalyzed formation of representative PG products.

Product	Pig Kidney Microsomes	COX-1		COX-2	
		*K*_m_ (μM)	*V*_max_ (nmol/mg/5 min)	*K*_m_ (μM)	*V*_max_ (nmol/mg/5 min)
**PGF_2α_**	-	4.5	25.0	ND	ND
	+	9.5	16.0	ND	ND
**PGE_2_**	-	ND	ND	1.2	47.7
	+	ND	ND	1.7	121.8
**PGD_2_**	-	ND	ND	1.0	33.2
	+	ND	ND	10.5	24.4
**12-HHT**	-	1.0	27.1	1.0	48.0
	+	4.0	74.9	4.3	165.5

ND: Not determined.
